# Summer Heat

**Published:** 1901-07-20

**Authors:** 


### Summer Heat.

The exceptionally great heat which has prevailed during the last few weeks in ordinarily temperate portions of the globe, and the distress and mortality which, especially in the United States, this great heat has occasioned, may be taken as another warning of the impossibility of fighting against the forces of nature, and of the necessity of evading rather than of opposing them. Many valuable lives have been lost from no other cause than the attempt to persevere, when the thermometer stood between 80° and 90°, in occupations which would have been easy and pleasant when it stood between 60° and 70°. Our own soldiers, sailors, and emigrants, in many tropical and subtropical regions, are constantly exposed to higher temperatures than those which have lately proved so fatal; but they avoid all ill-consequences by means of a careful adaptation of their hours and habits to the requirements of the circumstances which surround them. In these islands, where the normal variations of temperature keep well within extremes, and where such extremes as happen are never of more than brief duration, a certain degree of unpreparedness to meet them is not altogether surprising; but, in the United States, where the normal variations are far greater than with us, and where experience has shown that life must be rendered subject to them in August, it seems strange that a visitation of heat at a somewhat earlier period should have been met by resistance, rather than by that submission to the inevitable which wisdom would have dictated from the first, and which has been sadly proved to be the only way of safety. Hastily extemporised modifications of clothing have not always been judicious in their character, and the means adopted to quench thirst have probably left a very great deal to be desired.

For the great majority of people in this country it may fairly be said that the arrival of a temperature of 80° in the shade calls for some modification of the accustomed habits of life in respect alike of clothing, dietary, and work. In Holland the definite recognition of a similar requirement is secured to children, all schools being closed by law as soon as a certain temperature is reached and as long as it prevails. In respect of clothing, perhaps, the colour of garments is of as much importance as their texture. The power of woven fabrics to absorb and transmit heat is largely governed by their colour. No one who has not tried the experiment can realise the comfort that is derived, when the thermometer is at 80°, by exchanging the black coat and dark trousers of daily custom for garments of light grey or fawn colour, and the black "chimney-pot" hat for a white one. White linen jackets and white helmet covers would be an inexpressible boon to the police during high temperatures, especially to the men who are stationed at busy crossings to regulate the traffic, and who are necessarily exposed to the full power of the sun. Few things are more conducive to comfort under high temperatures than a careful regulation of the nature and quantities of the drinks that are consumed. To drink freely during extreme heat is merely to establish a vicious circle of imbibing and perspiring, and the more the drinker drinks the more thirsty will he become. If more drink be really required during hot weather than during cold, a position in favour of which it would perhaps be difficult to adduce any valid evidence, the increased quantity should not be taken during exertion, but during rest, and chiefly either in the morning before work is commenced or in the evening after it is finished. It is well known to campaigners that the men who drink on a march break down. Least of all, should a supposed requirement for an excess of drink be converted into an excuse for an excess of alcohol, which is certainly more prejudicial in hot weather than in cold. As regards solid food, a reduction in the quantity of its nitrogenous elements, and a corresponding increase in the proportion of rice, fresh vegetables, and fruit will unquestionably tend to the maintenance of healthy metabolism under the changed conditions. It would, of course, be eminently desirable if the necessary general consent could be obtained to submit to a modification of the ordinary hours of business throughout the continuance of an abnormal "heat wave," and to substitute a noonday rest for the feverish activity of mind and body to which we have become accustomed. In Great Britain, unless those are right who maintain that the local climate is gradually changing and the liability to excessive heat increasing, it seems improbable that our occasional visitations of high temperature will be sufficiently common and sufficiently declared to produce such a change in the national habits; but it seems quite clear that, in the United States, where the conditions to be provided against are more certain both to arrive and to remain than they are with us, some approach to the Dutch regulation about schools might with great advantage be applied to the docks and the counting houses of the great centres of business throughout the country. An abandonment of work in obedience to thermometric indications would in a few days have saved hundreds of lives.

